# Complement activation and effect of eculizumab in scleroderma renal crisis

**DOI:** 10.1097/MD.0000000000004459

**Published:** 2016-07-29

**Authors:** Arnaud Devresse, Selda Aydin, Moglie Le Quintrec, Nathalie Demoulin, Patrick Stordeur, Catherine Lambert, Sara Gastoldi, Yves Pirson, Michel Jadoul, Johann Morelle

**Affiliations:** a Division of Nephrology, Cliniques Universitaires Saint-Luc; b Institut de Recherche Expérimentale et Clinique, Université catholique de Louvain; c Division of Pathology, Cliniques Universitaires Saint-Luc, Brussels, Belgium; d Department of Nephrology and Transplantation, Lapeyronie Hospital, Montpellier, France; e Immunobiology Clinic, Université Libre de Bruxelles-Hôpital Erasme; f Hemostasis-Thrombosis Unit, Division of Hematology, Cliniques Universitaires Saint-Luc, Brussels, Belgium; g IRCCS – Istituto di Ricerche Farmacologiche “Mario Negri,” Clinical Research Center for Rare Diseases Aldo e Cele Daccò, Ranica, Bergamo, Italy.

**Keywords:** acute kidney injury, complement system, systemic sclerosis, thrombotic microangiopathy

## Abstract

**Background:**

Scleroderma renal crisis (SRC) is a life-threatening complication of systemic sclerosis characterized by abrupt onset of hypertension, thrombotic microangiopathy, and kidney injury. The mechanisms of the disease remain ill-defined, but a growing body of evidence suggests that activation of the complement system may be involved.

**Methods:**

Here, we report the case of a patient presenting with severe SRC and strong evidence of complement activation, both in serum and in the kidney, in the absence of genetic defect of the complement system.

**Results:**

Immunofluorescence studies on kidney biopsy showed significant deposits of C1q and C4d in the endothelium of renal arterioles, pointing toward activation of the classical pathway. Because of the dramatic clinical and histological severity, and the lack of response to early treatment with angiotensin-converting enzyme inhibitors, calcium channel blockers and plasma exchange, the patient was treated with the specific C5 blocker eculizumab.

Contrarily to conventional treatment, eculizumab efficiently blocked C5b-9 deposition ex vivo and maintained hematological remission. Unfortunately, the patient died from heart failure a few weeks later. Postmortem examination of the heart showed diffuse patchy interstitial fibrosis, the typical lesion of systemic sclerosis-related cardiomyopathy, but normal coronary arteries and myocardial microvasculature.

**Conclusion:**

SRC may lead to complement system activation through the classical pathway. Early administration of C5 inhibitor eculizumab may have therapeutic potential in patients with life-threatening SRC refractory to conventional treatment using angiotensin-converting enzyme inhibitors.

## Abstract 2

Supplemental Digital Content is available in the text

## 1 Introduction

Systemic sclerosis (SSc) is a complex autoimmune disorder characterized by microvascular damage and progressive fibrosis of the skin and visceral organs, especially the lungs, heart, and kidneys. Scleroderma renal crisis (SRC) occurs in ∼10% of SSc patients, and is characterized by abrupt onset of hypertension, thrombotic microangiopathy (TMA), and acute kidney injury.^[1]^ Although prognosis has improved with the use of angiotensin-converting enzyme inhibitors, 40% of patients still require dialysis, and 25% die within 1 year.^[2]^ The pathogenesis of SSc remains poorly understood but a growing body of evidence suggests that activation of the complement system may be involved in the disease. Here, we report the dramatic case of a young patient presenting with severe SRC during pregnancy in which complement activation was comprehensively documented both in serum and in the kidney, and effectively blocked by the specific C5 complement inhibitor eculizumab.

## 2 Case presentation

A 28 year-old female Caucasian patient was admitted to the emergency department at 28 weeks of a 1st twin pregnancy with hypertension (220/120 mm Hg), signs of TMA, and acute kidney injury (serum creatinine 2.67 vs 0.36 mg/dL 2 months earlier).

SSc was diagnosed 3 years earlier on the basis of an acrosyndrome, sclerotic skin changes, microvascular abnormalities on nailfold capillaroscopy, and significant titers of anticentromere (197 IU/L, normal <7) and anti-Scl70 (>240, normal <7 IU/L) antibodies. She was treated with nifedipine 30 mg od for hypertension. There was no family history of autoimmune disorder, kidney disease, or TMA. The pregnancy was hitherto uncomplicated, with no proteinuria and optimal blood pressure control.

**Figure 1:**
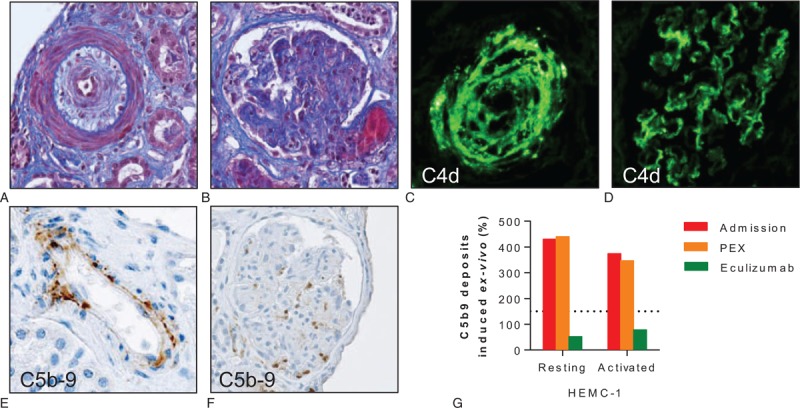
Scleroderma renal crisis and activation of the complement system. (A) Masson trichrome blue of a renal arteriole with intimal edema and onion-skin lesion narrowing the lumen (obj. ×25). (B) Masson trichrome blue of a glomerulus showing arteriolar thrombosis at the vascular pole. On light microscopy, most glomeruli appeared ischemic or even necrotic; in the remaining intact glomeruli, focal signs of mesangiolysis were present (obj. ×20). (C, D) C4d deposits identified by immunofluorescence in the wall of a renal arteriole (C) and glomerular capillaries (D). (E, F) Immunohistochemistry showing C5b-9 deposits in the endothelium of a renal arteriole (E) and a nonnecrotic glomerulus (F). (G) Ex vivo C5b-9 deposition induced by the serum of the patient. Relative surface area covered by C5b-9 staining after incubation of unstimulated (resting) or ADP-activated HMEC-1 for 4 hours with serum from the patient at admission (red bars), after 7 daily sessions of PEX (orange bars), and after 2 doses of eculizumab (green bars). C5b-9 deposits were normalized under eculizumab. Normal values <150% (dotted line). ADP = adenosine diphosphate, HMEC-1 = human microvascular endothelial cells-1, PEX = plasma exchange.

Lab tests at admission (Table 1) showed severe thrombocytopenia, microangiopathic hemolytic anemia, and ADAMTS13 activity in normal range (39%), ruling out thrombotic thrombocytopenic purpura. Liver function was unaltered, C3 and C4 complement levels were decreased, and urinalysis showed a bland sediment and gross proteinuria (4+). Cesarean delivery was performed on the day of admission because of TMA and fetal distress, and lisinopril 20 mg od and intravenous nicardipine were started. Daily plasma exchange had to be initiated 48 hours later because biological signs of TMA persisted and AKI had progressed to anuria, requiring dialysis initiation, making the diagnosis of preeclampsia unlikely. Systematic workup also ruled out HIV infection, antiphospholipid syndrome, and occult infection. Ultrasound showed kidneys of normal size, without thrombosis of renal arteries but with global bilateral hypoperfusion. Kidney biopsy showed severe vascular changes mainly in renal arterioles and, to a lesser extent, in glomerular capillaries (Fig. 1A, B). Light microscopy showed vascular lesions of intimal thickening by myxoid tissue, onion-skinning, fibrinoid necrosis, and intraluminal thrombosis in interlobular arterioles, along with extensive ischemic damage in the glomeruli and tubules. Eleven out of the 39 glomeruli appeared necrotic while the remaining ones presented signs of glomerular ischemia, thrombosis at the vascular pole, or mesangiolysis. Approximately 25% of the cortex was necrotic. Immunofluorescence studies identified deposits of C3 (2+), C1q (2+), C4d (3+) (Fig. 1C, D), and C5-b9 deposits were observed in the endothelium of renal arterioles and in glomeruli (Fig. 1E, F).

**Table 1:**
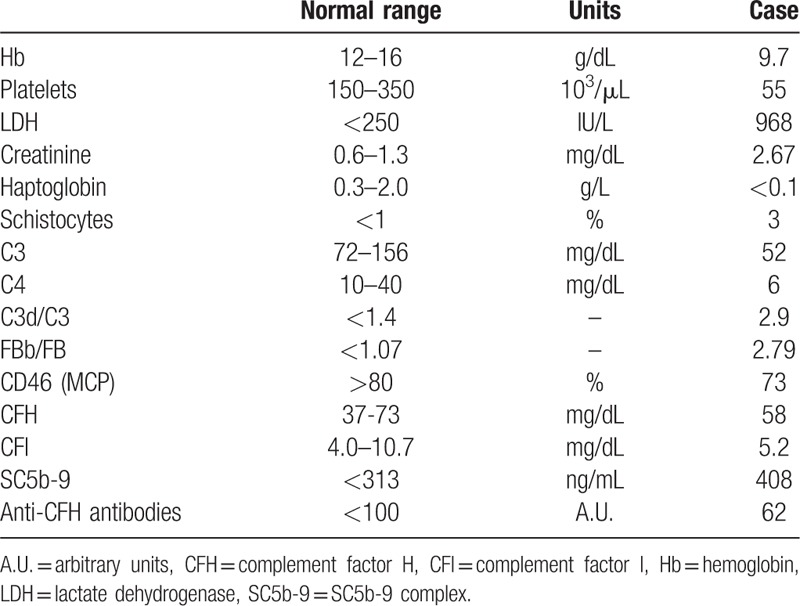
Biological data at admission.

Complement profile in the patient's serum sampled at admission showed, in addition to decreased levels of both C3 and C4, increased SC5b-9 levels, as well as high C3d/C3 and FBb/FB ratios (Table 1). Activation of the complement system was further confirmed by demonstrating the strong capacity of the patient's serum to induce C5b-9 deposits on cultured human microvascular endothelial cells ex vivo (Fig. 1G).^[3]^ An extensive genetic screening found no mutation in the genes encoding the components of the alternative pathway of complement or its regulatory proteins known to be involved in atypical hemolytic uremic syndrome (aHUS), including *CFH*, *CFI*, *MCP*, *C3*, *CFB*, *CFH*-related proteins and *THBD*, and anti-CFH antibodies were not detected.

Because of the dramatic clinical and histological severity of the SRC and several lines of evidence supporting complement activation, eculizumab was initiated 18 days after admission (4 weekly 900 mg infusions, followed by 1200 mg every 2 weeks) (Supplementary Fig. 1). Despite prompt and optimal control of blood pressure, normalization of volume status with intermittent hemodialysis, and effective blockade of the complement system by the C5 inhibitor (Fig. 1G), serum troponin T levels increased progressively (Supplementary Table 1) and the patient developed new onset heart failure (left ventricle ejection fraction ∼22%). There was no evidence of pulmonary arterial hypertension. The patient rapidly developed acute pulmonary edema, supraventricular tachycardia followed by bradyarrhythmia and cardiac arrest. Despite early cardiopulmonary resuscitation, pacing, and an attempt of extracorporeal membrane oxygenation, the patient died from heart failure 8 weeks after the onset of the SRC. At autopsy, coronary arteries and myocardial microvasculature appeared normal (i.e., with no sign of TMA or atherosclerotic disease). The pericardium was normal, but the left ventricle showed diffuse mild patchy interstitial fibrosis (Supplementary Fig. 2), the typical lesion of the most severe forms of SSc-related cardiomyopathy.^[4]^

## 3 Discussion

This patient presented a severe SRC during pregnancy, with extensive lesions of TMA in the kidney. The strong evidence of systemic and local, intrarenal, activation of the complement system suggests it has played a role in the pathogenesis of kidney injury. Treatment with the specific C5 blocker eculizumab was associated with hematological remission of the disease and a sharp decrease in C5b-9 deposits ex vivo.

The complement system is part of innate immunity, acting as a 1st-line defense against pathogens and maintaining host homeostasis. Complement activation initiates a cascade reaction that generates bioactive components (C3b, C3a, C5a, and C5b-9) with proinflammatory, chemo-attractant, and cell-damaging functions.^[5]^ A few observations suggested that activation of the complement system might be involved in the pathophysiology of SSc and SRC. First, C3 and/or C4 hypocomplementemia is present in ∼15% of patients with SSc, and associated with disease severity and vascular involvement.^[6,7]^ Second, C5b-9 deposition was detected in capillaries of skin biopsies of SSc patients but not in healthy subjects.^[8]^ Third, C4d deposition was found in renal peritubular capillaries of a subset of SSc patients with a poor renal outcome.^[9]^ In our patient, systemic complement activation during severe SRC was unequivocally demonstrated by a constellation of findings: hypocomplementemia; circulating complement profile with increased levels of SC5b-9 and increased C3d/C3 and FBb/FB ratios;^[10]^ vascular deposition of C5b-9 in injured glomerular capillaries and renal arterioles; and the capacity of the patient's serum obtained at admission to induce SC5b-9 deposits on cultured microvascular endothelial cells.^[3]^ These findings were reminiscent of those observed in patients with complement-mediated aHUS, the prototype of diseases resulting from ineffective protection of the endothelium from complement attack.^[11]^ Altogether they suggested that abnormal activation of the complement system may be involved in the pathogenesis of SRC.

Thorough assessment of complement fractions deposition in damaged vessels also provided clues to the mechanism underlying complement activation in this setting. Basically, 3 different pathways (classical, lectin, and alternative) can activate the complement system. They differ in their recognition target: antibodies in the classical pathway, carbohydrate residues on microorganisms in the lectin pathway, and a permanent low-level activation in the alternative pathway.^[12]^ Activation of the classical pathway has been well documented in antibody-mediated rejection and lupus erythematosus, that is, through the C1 component and its pattern recognition molecule C1q.^[5,13–15]^ In these situations, the deposition of C4d – a byproduct of activation of the classical and lectin pathways – in injured tissues is informative of the mechanism by which the complement system is activated and has been associated with disease severity and poor outcome.^[16–19]^ In our patient, the coexistence of intense C4d and C1q deposits in renal arteries and glomerular capillaries demonstrated that complement activation in SRC occurred through the classical pathway. Although it is tempting to speculate that autoantibodies or immune complexes might be responsible for such activation during SRC, we cannot formally rule out the effect of hypertension-induced hemodynamic shear-stress, which has also been shown to activate the classical pathway of the complement system on endothelial cells.^[20]^ The increased FBb/FB ratio found in patient's serum suggested subsequent recruitment of the alternative pathway through the C3b feedback cycle, as previously described in lupus erythematosus.^[21]^

Complement inhibition – on top of blockade of the renin–angiotensin system – might be useful in severe refractory cases of SRC, when complement activation has been carefully documented.^[22]^ The efficiency of eculizumab – a recombinant, fully humanized, monoclonal antibody directed against the human complement component C5 – was demonstrated in patients with aHUS.^[23]^ More recent evidence also suggested it might be useful in diseases driven by complement activation through the classical pathway, such as antibody-mediated rejection^[24]^ and lupus nephritis.^[25,26]^ In these conditions confirmatory evidence is however awaited and clinical trials are in progress. In our patient, eculizumab efficiently blocked C5b-9 deposition ex vivo, contrarily to plasma exchange, and maintained hematological remission. The lack of recovery of renal function despite both effective C5 blockade and prompt blood pressure control using angiotensin-converting enzyme inhibitors is likely explained by the extent of preexisting cortical necrosis. The development of heart failure under complement inhibition and the absence of overt myocardial vascular lesions at postmortem examination suggested an independent pathophysiological process, possibly related to abnormal vasoreactivity.^[4]^

In conclusion, we report a fatal case of SRC in which several lines of evidence demonstrated complement activation through the classical pathway. Further studies are needed to decipher the mechanisms of complement activation in SSc and its role in the pathogenesis of SRC, and to confirm the potential benefit of early eculizumab administration in patients with refractory, life-threatening SRC despite the use of angiotensin-converting enzyme inhibitors.

## 4 Ethics committee

The Ethics Committee of the Cliniques Universitaires Saint-Luc approved this case-report and encouraged its publication despite the absence of written informed consent from the patient – who died during the course of the disease – as the case-report ensures complete confidentiality and respect of private life, in agreement with national laws. In addition, all investigations were performed based on clinical needs.

## Acknowledgements

The authors thank Fondation Saint-Luc (JM), the Fondation Horlait-Dapsens (JM), and the Fonds National pour la Recherche Scientifique (JM) and the European Union FP7 EURenOmics Project number 305608 (SG) for the support.

## Supplementary Material

Supplemental Digital Content
