# Supplementary material for: Complement activation and effect of eculizumab in scleroderma renal crisis
Source: Medicine (Baltimore). 2016 Jul 29;95(30):e4459. doi: 10.1097/MD.0000000000004459 (PMC5265879; doi:10.1097/MD.0000000000004459)
Supplement: Supplemental Digital Content [file medi-95-e4459-s001.docx]

MD-D-1601951_R2

**SUPPLEMENTARY MATERIAL**

**Complement Activation and Effect of Eculizumab
in Scleroderma Renal Crisis**

***Case Report and Literature Review***

Arnaud Devresse, MD, Selda Aydin, MD, Moglie Le Quintrec, MD, PhD, Nathalie Demoulin, MD, Patrick Stordeur, PhD, Catherine Lambert, MD, Sara Gastoldi, MD, Yves Pirson, MD, Michel Jadoul, MD, Johann Morelle, MD

***Table of content***

*Methods P 2*

*References P 3*

**methods**

***Immunofluorescence staining.*** For immunofluorescence, 3 μm cryostat sections were stained with fluorescein isothiocyanate (FITC)-conjugated polyclonal rabbit antihuman C1q (Code no. F 0254, Dako) at 1:20 dilution (direct immunofluorescence) and, a murine monoclonal anti-human C4d (cat. No. A213, Quidel) at 1:100 dilution with secondary fluorescein isothiocyanate (FITC)-conjugated polyclonal rabbit anti-mouse IgG (Code no. F 0232, Dako) at 1:20 dilution (indirect immunofluorescence). The intensity of immunofluorescence positivity was graded on a scale of 0, trace, and 1 to 3+.

***Staining of C5b9.*** Immunohistochemistry was performed from paraffin-embedded kidney sections as previously described (1). Sections were analyzed for deposits C5b9 neoantigen (gift from Professor Paul Morgan Cardiff, UK) as primary antibody diluted to 1:50, the biotinylated anti-mouse IgG secondary antibody diluted to 1:300 was performed and streptavidine-biotin-peroxydase complex system was used for signal amplification.

***Testing of the complement system.*** C3, C3d, CFH, CFI and FB were quantified by nephelometry on a Siemens BNII instrument. Measurement of C3 and FB was performed on plasma following manufacturer’s instructions, while CFH and CFI were analyzed on serum following home-made protocols available on request. After separation of plasmatic C3d from native C3 by polyethylene glycol precipitation, the C3d was quantified in the precipitate by nephelometry on the same instrument, adapted from a previously described method (2). FBb and SC5b-9 complex were quantified in plasma by ELISA methods using commercial kits (MicroVue SC5b-9 Plus EIA, MicroVue Bb Plus EIA, Quidel, San Diego, CA 92121) according to the manufacturer’s instructions. An ELISA was also used to detect anti-CFH antibodies, as previously described (3).

***Genetic testing of the genes encoding the components of the alternative pathway of complement or its regulatory proteins.*** Genetic testing was performed using SALSA MLPA probemix P236 to detect deletions/duplications of one or more sequences in the *CFH* and *CFH*- related (*CFHR*) genes; DNA sequencing based on Sanger method for the *CFH, CFI* and *MCP* genes; a diagnostic panel for all known genes encoding complement regulatory proteins (this test is based on a combination of multiplex PCR and high throughput sequencing -next-generation sequencing).

***Membrane expression of CD46 (MCP).*** One hundred µL of EDTA whole blood were incubated during 20 minutes with saturating concentrations of fluorescein-conjugated antibody against MCP (clone E4.3; BD Pharmingen) at room temperature. After lysis of red blood cells with the BD Facs Lysing Solution (BD Biosciences, Mountain View, CA), acquisition was performed using a FacsCalibur flow cytometer and data were analyzed using BD CellQuest software (BD Biosciences). MCP expression was analyzed on granulocytes that were identified on basis of forward versus side-scatter. Results were expressed both in percentages of MCP expression compared to controls.

***Ex vivo C5b-9 deposition.*** C5b-9 deposition induced by the patient serum was assessed *ex vivo*, as previously described (4). Briefly, the serum was incubated during 4 hours on cultured microvascular endothelial cells (HMEC-1), in resting conditions and after activation by adenosine5’-diphospate (ADP). SC5b-9 deposition was then assessed using immunofluorescence and confocal microscopy.

***Picrosirius red staining.*** Five µm sections were deparaffinized, rehydrated, treated with 1% phosphomolybdic acid and incubated in saturated picric acid solution containing 1% sirius red for 2h at room temperature before washing in 0.01N hydrochloric acid and mounting. Samples were scanned using a SCN400 slide scanner (Leica, Heerbrugg, Switzerland).

**REFERENCES**

1. Le Quintrec M, Lionet A, Kandel C, et al. Eculizumab for treatment of rapidly progressive C3 glomerulopathy. *Am J Kidney Dis* 2015;65:484-489.
2. Vergani D, Bevis L, Nasaruddin BA, Mieli-Vergani G, Tee DE. Clinical application of a new nephelometric technique to measure complement activation. *J Clin Pathol* 1983;36(7):793-797.
3. Watson R, Lindner S, Bordereau P, et al. Standardisation of the factor H autoantibody assay. *Immunobiology* 2014;219(1):9-16.
4. Noris M, Galbusera M, Gastoldi S, et al. Dynamics of complement activation in aHUS and how to monitor eculizumab therapy. *Blood* 2014;124(11):1715-1726.

**SUPPLEMENTARY TABLE**

**Supplementary table 1. Biological data during follow-up.**

|  | **Normal range** | **Units** | **Time (days from admission)** | | | | | | | |
| --- | --- | --- | --- | --- | --- | --- | --- | --- | --- | --- |
|  |  |  | **1** | **5** | **10** | **15** | **20** | **30** | **40** | **50** |
| **Platelets** | 150-350 | 10³/µl | 55 | 68 | 109 | 174 | 338 | 214 | 171 | 158 |
| **LDH** | <250 | IU/l | 968 | 777 | 621 | 558 | 320 | 318 | 341 | 412 |
| **Haptoglobin** | 0.3-2.0 | g/l | <0.1 | <0.1 | <0.1 | 0.1 | 1.1 | 0.6 | 1.11 | 1.0 |
| **Schistocytes** | <1 | % | 3 | 3 | 5 | 4 | 4 | <1 | <1 | 2 |
| **TnT** | <15 | pg/ml | 454 | 580 | 880 | 927 | 623 | 704 | 921 | 1030 |
| **Hb** | 12-16 | g/dl | 9.7 | 7.9 | 7.5 | 5.7 | 7.7 | 7.3 | 7.3 | 7.7 |

LDH, lactate dehydrogenase; TnT, troponin T levels; Hb, hemoglobin

Figure S1: Evolution of platelets level and lactate dehydrogenase (LDH) during plasma exchanges and after initiation of eculizumab

Figure S2: Post-mortem examination of the heart

(A-B) Picrosirius red-stained sections of the left ventricle showing the accumulation of collagen fibers in the case (B) as compared with a gender- and age-matched control patient (A), with no history of SSc. Original magnification 10x, bar, 100 µm. (C-D) Coronary arteries with no evidence of macrovascular lesion, both in the patient (D) and the control (C). Original magnification 2x, bar, 1 mm.


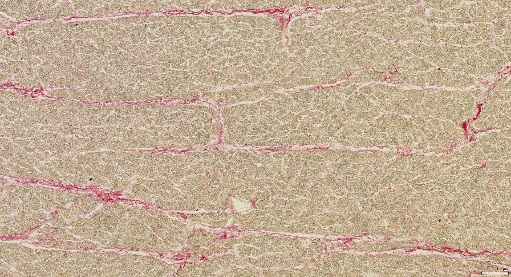

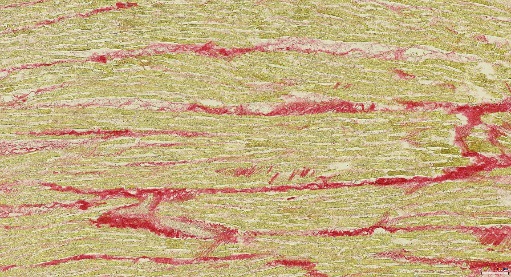


Control

Case

Left ventricle


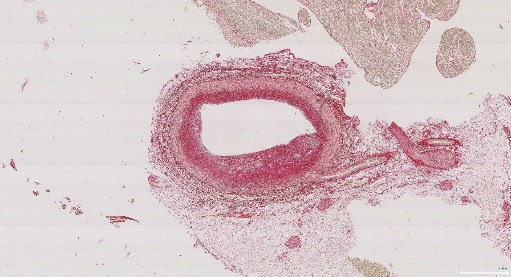

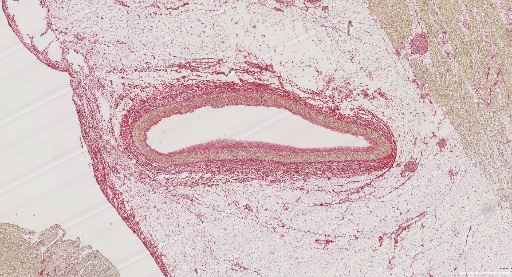


Coronary artery

1 mm

1 mm

100 µm

100 µm

A

B

C

D
